# Atypical primary pulmonary amyloidosis: A rare case report

**DOI:** 10.1097/MD.0000000000020828

**Published:** 2020-06-26

**Authors:** Xiong Peng, Xiaolei Wang, Daya Luo, Wei Zuo, Huiming Yao, Wei Zhang

**Affiliations:** a Department of Respiratory and Critical Care Medicine, The First Affiliated Hospital of Nanchang University; b Second Department of Cardiovascular Medicine, Jiangxi Provincial People's Hospital Affiliated of Nanchang University; c Department of Biochemistry and Molecular Biology, The Basic Medical School of Nanchang University, Nanchang, Jiangxi, China.

**Keywords:** case report, computed tomography, pulmonary amyloidosis, treatment

## Abstract

**Rationale:**

Pulmonary amyloidosis is a rare respiratory disease characterized by amyloid deposition in the lungs. The clinical manifestations of pulmonary amyloidosis are variable and without specific symptoms.

**Patient concerns:**

We report a rare case of tracheobronchial amyloidosis to improve our understanding of the disease.

**Diagnoses:**

The diagnosis of tracheobronchial amyloidosis was finally established by transbronchoscopic lung biopsy and histological examination.

**Interventions:**

The patient significantly improved with methylprednisolone sodium succinate for injection (40 mg/day) for 5 days and low-dose oral prednisone for 10 days.

**Outcomes:**

After treatment, discomfort, such as cough, stridor, dyspnea, and chest tightness, disappeared, and he was discharged. The patient was in good clinical condition after 8 months of follow-up.

**Conclusion:**

This case clearly shows that it is difficult to distinguish tracheobronchial amyloidosis from other diseases with manifestations of cough, dyspnea and chest tightness because of their similar symptoms and imaging findings. Thus, the role of transbronchoscopic lung biopsy and histological examination in the diagnosis of tracheobronchial amyloidosis is very important.

## 1 Introduction

Lungs and bronchi can be affected by systemic and localized amyloidosis. Systemic amyloidosis is listed among rare diseases with an estimated incidence of less than 10 cases per million person-years and are caused by conformational changes and the aggregation of autologous proteins that deposit in tissues in the form of fibrils.^[1]^ Localized amyloidosis, such as primary pulmonary amyloidosis, is a more uncommon disease characterized by the extracellular deposition of insoluble fibrillar proteins localized to the respiratory system.^[1,2]^ This was originally described in an autopsy specimen by Lesser in 1877.^[3]^ There are 3 different forms of pulmonary amyloidosis: tracheobronchial amyloidosis, diffuse alveolar-septal amyloidosis and nodular pulmonary amyloidosis.^[4]^ Among these, tracheobronchial amyloidosis is the least common form of pulmonary amyloidosis, and nearly 100 such cases have been reported. The median age of patients is between 50 and 60 years, and there is no sex predilection.^[5,6]^ The manifestations and prognosis of pulmonary amyloidosis vary considerably depending on its etiology and anatomical distribution.^[7]^ Patients usually present with cough, dyspnea, pleural effusions, hoarseness, or hemoptysis.^[8]^ Bronchoscopy and histological examination are crucial for establishing the diagnosis of tracheobronchial amyloidosis. Furthermore, chest computed tomography (CT) is very useful for determining the extent of this disease.^[9,10]^ Although the number of reported cases has accumulated over past years, the exact pathogenesis of the disease remains unclear. It is believed that the misfolding of extracellular proteins plays a critical role in the molecular mechanism of pulmonary amyloidosis. Therefore, the management of pulmonary amyloidosis is difficult and largely dependent upon symptoms. In addition, to date, there is no proven drug therapy for tracheobronchial amyloidosis. Recent studies report that systemic chemotherapy may benefit patients with progressive disease and that invasive bronchoscopic therapies, such as intermittent bronchoscopic resection, bronchoscopic Nd:YAG laser debulking, and even surgical resection, may be useful for relieving airway obstruction and improving uncomfortable symptoms.^[11–14]^ Primary pulmonary amyloidosis in the tracheobronchial airways that is not associated with systemic amyloidosis is very rare. Here, we report a case of tracheobronchial amyloidosis without systemic involvement.

## 2 Case presentation

**Figure 1:**
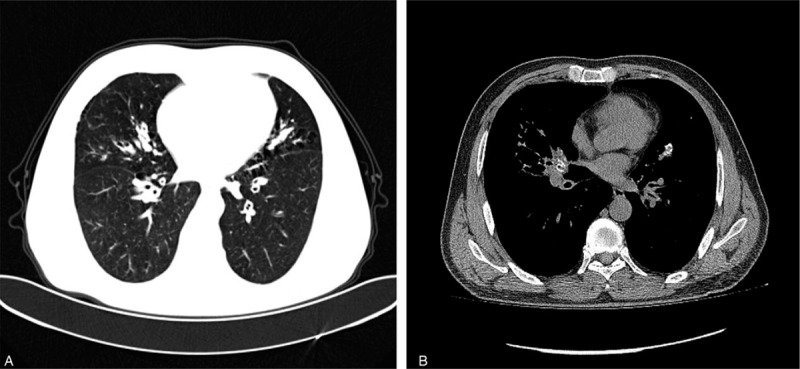
High-resolution chest CT showing right lung middle lobe atelectasis with diffused calcification and a thick-walled cavity in the bilateral bronchi (1A and 1B).

A 59-year-old Chinese man was referred to the Departments of Respiratory Medicine, The First Affiliated Hospital of Nanchang University, Nanchang, in July 2019 due to a 6-month history of repeated cough, stridor, dyspnea, and chest tightness without other symptoms such as chest pain, fever, nausea, hemoptysis or vomiting. He denied a history of smoking, dust exposure, lung disease connective tissue disorders or malignancy. His physical examination was unremarkable. The results of laboratory tests showed that the data for a peripheral blood count, baseline serum chemistry screening, urinalysis, stool examination, a purified protein derivative test for tuberculosis and tumor biomarker tests were all normal as were electrocardiogram, echocardiography and abdominal ultrasound. A CT of the chest revealed right lung middle lobe atelectasis with diffused calcification and a thick-walled cavity in the bilateral bronchi. Fibrous substances were deposited in the bronchopulmonary tissues (Fig. 1A, 1B). In addition, a bone scan and magnetic resonance scan of the brain were performed, and all images were normal.

**Figure 2:**
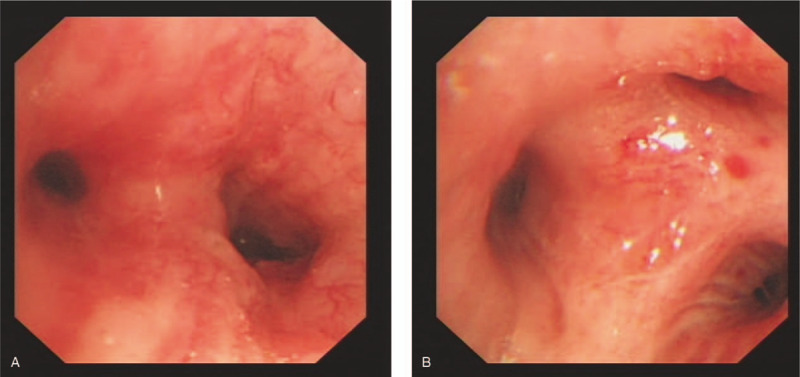
Bronchoscopic image of tracheobronchial amyloidosis, showing mild stenosis of the trachea (2A) and submucosal deposits (2B).

**Figure 3:**
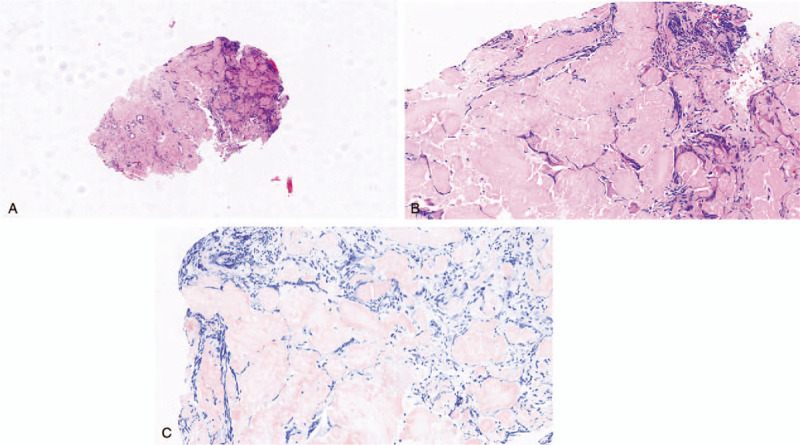
3A, At low magnification, amyloid deposits surround seromucous glands and cartilage plates. 3B, Higher magnification shows eosinophilic deposits, a lymphoplasmacytic infiltrate, and ossification. 3C, Congo red-stained amyloid deposits were deep-pink under the microscope. (hematoxylin-eosin, original magnifications ×40 [3A] and × 200 [3B]) (Congo red staining, original magnifications × 200[3C]).

Finally, the patient underwent a bronchoscopic examination of the airway. The results showed that the bronchial mucosa was slightly bulged, which caused mild stenosis of the trachea, and a large amount of whitish powdery homogeneous substance deposition was observed between the mucosal glands, which revealed tracheobronchial infiltration and parenchymal infiltration (Fig. 2A, 2B). A biopsy was taken. A histological examination showed that the specimens contained amorphous acidophilic homogeneous material with some plasmocytes, lymphocytes, fibroblasts and giant cells. Eosinophilic material displayed green birefringence under polarizing microscopy. Immunohistochemical staining suggested CD31 (blood vessel) and CD34 (blood vessel) positivity. Moreover, Congo red staining was positive, and Periodic Acid-Schiff staining was positive, confirming the deposition of amyloid within the specimen (Fig. 3A, 3B, 3C). Therefore, a diagnosis of primary tracheobronchial amyloidosis was established.

The patient was treated with methylprednisolone sodium succinate for injection (40 mg/day) for 5 days. After that, he was treated with low-dose oral prednisone for 10 days. Then, discomfort, such as cough, stridor, dyspnea, and chest tightness, disappeared, and he was discharged. The patient was in good clinical condition after 8 months of follow-up (from July 19, 2019 to March 19, 2020). However, diffuse calcification and the thick-walled cavity in the bilateral bronchi showed no changes and were confirmed by a reviewed CT scan.

## 3 Discussion and conclusion

Amyloidosis is a disorder caused by misfolding of autologous protein and its extracellular deposition as fibrils, resulting in vital organ dysfunction and eventually death.^[15]^ Based on immunohistochemistry, there are 4 major categories of amyloidosis: primary or immunoglobulin light chain (AL) disease, hereditary or mutant transthyretin disease, secondary or amyloid protein A disease and dialysis-associated or β2 -microglobulin disease.^[16,17]^ In particular, AL fibril is a common type of amyloid deposit in the lung. Pulmonary amyloidosis is a rare disease and may be localized or part of systemic amyloidosis.^[1]^ Furthermore, pulmonary amyloidosis mainly comes in 3 forms according to the pathologists perspective; these include nodular pulmonary amyloidosis, diffuse alveolar-septal amyloidosis and tracheobronchial amyloidosis.^[4]^ Nodular pulmonary amyloidosis is usually localized and usually presents with peripheral subpleural localizations of variable size under chest CT. It is defined as one or more nodular amyloid deposits involving the lung, which usually represents as localized AL or AL/AH (mixed immunoglobulin light/heavy chain) amyloidosis.^[15,18]^ Diffuse alveolar-septal amyloidosis is usually a manifestation of systemic amyloidosis, is associated with systemic AL amyloidosis and is characterized by the presence of amyloid deposits in the vessel walls and alveolar septa.^[16,19]^ Tracheobronchial amyloidosis is an organ-limited type of amyloidosis that mainly represents multifocal submucosal plaques and usually presents localized AL amyloidosis without pulmonary parenchyma. Generally, it is symptomatic due to stenosis resulting from the amyloid deposits in the trachea and large bronchi.^[5,20]^ Patients usually present typical symptoms, such as dyspnea, cough, chest tightness, and even hemoptysis. On pulmonary function tests, subglottic amyloidosis may cause fixed airflow obstruction on spirometry. Tracheal and bronchial wall thickening with calcification or ossification is usually observed on a CT scan, and this may help indicate the diagnosis.^[21]^ Tracheobronchial endoscopy usually shows irregular diffuse whitish deposits and tracheal stenosis. Because the lesions are very fragile, they may bleed after biopsy. Deposits are localized to blood vessels and the submucosa, which are often associated with giant cells and plasma cells.^[2,14]^ In our case, the amyloid was mainly deposited in the tracheobronchial airways without systemic deposits. Our patient presented with repeated cough, dyspnea and chest tightness. A chest CT scan revealed right lung middle lobe atelectasis with diffused calcification and a thick-walled cavity in the bilateral bronchi, and a bronchoscopic examination showed that the bronchial mucosa was slightly bulged, which caused mild stenosis of the trachea, and a large amount of whitish powdery homogeneous substance deposition was observed between the mucosal glands. Finally, pulmonary amyloidosis was diagnosed based on histological examination. To summarize, the characteristics of this patient were completely consistent with tracheobronchial amyloidosis.

The clinical manifestations of pulmonary amyloidosis are variable. The majority of patients may present with tracheobronchial infiltration, parenchymal infiltration, persistent pleural effusions, or pulmonary hypertension.^[4,8,15]^ For example, in our case, the patients airway was narrow, and the amyloid deposits severely affected gas exchange alveolar structure, thus resulting in serious respiratory impairment. Tissue biopsy was necessary, and green-yellow-orange birefringence under polarized light after Congo red staining is the gold standard for the diagnosis and typing of pulmonary amyloidosis,^[22]^ which is very important and helpful for differentiating it from other diseases. Therefore, the differential diagnosis of pulmonary amyloidosis is broad and mainly includes infectious, interstitial, neoplasms, pulmonary sarcoidosis, interstitial, and granulomatous lung diseases.^[19,22]^

Because each patient may show different symptoms, we first need to identify the forms of pulmonary amyloidosis according to manifestations and a chest CT before initiating treatment. For instance, nodular pulmonary amyloidosis is usually an incidental finding on CT scan and often has no symptoms. Generally, nodular amyloidosis is treated satisfactorily by conservative excision, and the prognosis is excellent.^[6]^ Diffuse alveolar-septal amyloidosis is associated with systemic AL amyloidosis and is usually treated based on the underlying systemic amyloidosis. However, there are no specific data about the impact of treatment of systemic AL amyloidosis on pulmonary involvement.^[16,19,23]^ Moreover, tracheobronchial involvement with resultant stenosis is the main symptomatic presentation in tracheobronchial amyloidosis. Management of tracheobronchial amyloidosis depends largely on symptoms. It is said that bronchoscopic interventions (such as carbon dioxide laser ablation or bronchoscopic Nd:YAG laser debulking) or external beam radiation therapy may benefit patients.^[12–14]^ We can only resolve the patients symptoms of discomfort, and there are no systemic chemotherapy or bronchoscopic interventions. Fortunately, the patient was in good clinical condition during the follow-up period. There are 2 main characteristics of this case. First, our patient presented with repeated cough, dyspnea, and chest tightness, which may, for a long time, be falsely diagnosed as asthma,^[24]^ and second, tracheobronchial amyloidosis is usually treated with bronchoscopic interventions or external beam radiation therapy. However, our patient recovered without using the 2 methods above. Therefore, we want to consider this case as an atypical primary pulmonary amyloidosis.

Prognosis of pulmonary amyloidosis varies according to forms of them and individuals. Because most cases of nodular pulmonary amyloidosis are localized, and conservative excision is very useful, the long-term prognosis is excellent.^[6]^ Baumgart JV et al reported 183 patients with AL amyloidosis and found that nodular pulmonary amyloidosis showed a significantly better disease-specific 10-year survival compared with systemic amyloidosis (96.0% vs 51.9%).^[1]^ Since primary diffuse alveolar septal amyloidosis is extremely rare and few case series were published earlier.^[10,24]^ The average survival period in patients is 16 months only.^[23]^ Recently, Gandham et al reported a patient of diffuse alveolar septal amyloidosis, who was treated with chemotherapy and was well with symptomatic improvement in the 6-month follow-up.^[25]^ Finally, for the tracheobronchial amyloidosis, it was reported that, in some cases, the estimated 5 years survival rate was 30% to 50% with the treatment of radiotherapy.^[6]^ Neben-Wittich et al reported 7 consecutive patients who were treated with external beam radiation therapy, and only 1 patient achieved an objective improvement as well as asymptomatic response in terms of CT scan results and bronchoscopic findings after 17 months of follow-up.^[13]^ Moreover, O’Regan et al. reported ten patients referred to the Amyloid Program at Boston University over the past 15 years and found that 30% of tracheobronchial amyloidosis patients died within 7 to 12 years after diagnosis and all having proximal or severe mid airways disease.^[20]^ In general, the long-term prognosis of nodular pulmonary amyloidosis is the best, but diffuse alveolar septal amyloidosis is the worst.

Overall, amyloidosis of the lower respiratory tract is very rare but may reflect a significant clinical problem in either organ-limited or systemic amyloidosis. The most fundamental treatment options for amyloidosis are based on the fibril protein type. Targeting different pathogenic mechanisms, such as the unequivocal identification of the amyloid type, is vital to avoid therapeutic errors.^[14,22]^ Therefore, to achieve precise treatment, all new patients require unequivocal amyloid typing and complete assessment to determine their optimal treatment. Although there is still no proven drug therapy for tracheobronchial amyloidosis, luckily, several new drugs that can accelerate the clearance of tissue amyloid deposits, interfere with amyloid fibrillogenesis and stabilize the amyloid precursor proteins in the pipeline could potentially benefit patients with pulmonary amyloidosis.

## Acknowledgments

The authors thank the National Natural Science Foundation of China (81160248 and 81560464) and Natural Science Foundation of Jiangxi Province (20151BAB205058) for the support.

## Author contributions

**Conceptualization:** Xiong Peng, Daya Luo.

**Methodology:** Xiong Peng, Xiaolei Wang.

**Resources:** Wei Zuo, Huiming Yao.

**Supervision:** Xiong Peng, Daya Luo, Huiming Yao.

**Writing – original draft:** Xiong Peng.

**Writing – review & editing:** Wei Zhang.
